# Quality Assurance of Teleconsultations in a Store-and-Forward Telemedicine Network – Obtaining Patient Follow-up Data and User Feedback

**DOI:** 10.3389/fpubh.2014.00247

**Published:** 2014-11-24

**Authors:** Richard Wootton, Joanne Liu, Laurent Bonnardot

**Affiliations:** ^1^Norwegian Centre for Integrated Care and Telemedicine, University Hospital of North Norway, Tromsø, Norway; ^2^Faculty of Health Sciences, University of Tromsø, Tromsø, Norway; ^3^Médecins Sans Frontières (MSF) International, Geneva, Switzerland; ^4^Department of Medical Ethics and Legal Medicine (EA 4569), Paris Descartes University, Paris, France; ^5^Fondation Médecins Sans Frontières, Paris, France

**Keywords:** telemedicine, telehealth, quality assurance, quality control, LMICs

## Abstract

User surveys in telemedicine networks confirm that follow-up data are essential, both for the specialists who provide advice and for those running the system. We have examined the feasibility of a method for obtaining follow-up data automatically in a store-and-forward network. We distinguish between *follow-up*, which is information about the progress of a patient and is based on outcomes, and user *feedback*, which is more general information about the telemedicine system itself, including user satisfaction and the benefits resulting from the use of telemedicine. In the present study, we were able to obtain both kinds of information using a single questionnaire. During a 9-month pilot trial in the Médecins Sans Frontières telemedicine network, an email request for information was sent automatically by the telemedicine system to each referrer exactly 21 days after the initial submission of the case. A total of 201 requests for information were issued by the system and these elicited 41 responses from referrers (a response rate of 20%). The responses were largely positive. For example, 95% of referrers found the advice helpful, 90% said that it clarified their diagnosis, 94% said that it assisted with management of the patient, and 95% said that the telemedicine response was of educational benefit to them. Analysis of the characteristics of the referrers who did not respond, and their cases, did not suggest anything different about them in comparison with referrers who did respond. We were not able to identify obvious factors associated with a failure to respond. Obtaining data by automatic request is feasible. It provides useful information for specialists and for those running the network. Since obtaining follow-up data is essential to best practice, one proposal to improve the response rate is to simplify the automatic requests so that only patient follow-up information is asked for, and to restrict user feedback requests to the cases being assessed each month by the quality assurance panel.

## Introduction

Follow-up is an integral part of consultation in medical practice. No doctor would give advice about a patient without attempting to follow the patient’s subsequent progress and/or trying to obtain some feedback. This basic principle is not altered when the consultation takes place at a distance (teleconsultation). Follow-up is part of routine clinical care, conducted in order to confirm that the situation is evolving as expected, and to allow the diagnosis, prognosis, and treatment to be adjusted as appropriate. It is also important for doctors to learn from their successes and mistakes, as part of a reflective practice (1).

Thus, it is not surprising that surveys in telemedicine networks show that the specialists who provide advice wish to receive follow-up data about the cases they have worked on. In a survey of telemedicine users in Médecins Sans Frontières (MSF), almost all specialists wanted follow-up information (52% considered follow-up desirable and 47% considered it necessary or mandatory) (2). In a survey of specialists in the Swinfen telemedicine network, 83% stated that they would like to receive follow-up information about the patient (3). We assume that provision of follow-up data is useful in keeping the specialists motivated, i.e., to ensure their continued participation in the telemedicine network and their availability to provide advice. It is also probably the only way that specialists can improve their service, since many of them will be based in high-income countries and without feedback it is impossible for them to know if their answers are useful; prompt feedback from the referrer may be perceived as a mark of gratitude for the service provided, which is important since many specialists volunteer their time and expertise for free. While it can reasonably be assumed that the provision of follow-up data is useful for many reasons, there is no literature about this (an experiment to test the assumption would be difficult, although not impossible).

Follow-up is also useful for those running the network, especially if a research study is to be conducted. Follow-up provides information about the value of the telemedicine consultations, and about the performance of individual specialists. Information about the latter is very valuable for the case coordinator in the allocation process, since experience shows that some specialists answer more quickly and comprehensively than others. Finally, providing follow-up data is probably a good discipline for the referrers, as it makes them think about the progress of their patients and about the value of the telemedicine advice they have received.

In the present paper, we distinguish between patient *follow-up*, which is information about the progress of a patient and is based on outcomes, and user *feedback*, which is more general information about the telemedicine system itself, including user satisfaction and the benefits resulting from the use of telemedicine.

### Objectives

The primary research question was whether a method could be developed for obtaining follow-up data automatically in a general teleconsulting network, which was providing a service in low-resource settings. The secondary research question was whether it was feasible to obtain both follow-up data and user feedback simultaneously.

## Methods

The present study required the development of a method to obtain data from the referrers and then a demonstration of its feasibility in practice. We combined the collection of both kinds of information into a single questionnaire, i.e., it represented a progress report.

The work was performed in two stages:

1. development of an information-collection tool;
2. demonstration of its feasibility in the MSF telemedicine network. Details of the network have been published elsewhere (2, 4).

Ethics permission was not required, because patient consent to access the data had been obtained and the work was a retrospective chart review conducted by the organization’s staff in accordance with its research policies.

### Development of the questionnaire

A questionnaire was developed by a consensus between three experienced telemedicine practitioners (two were medical specialists with field experience). It was based on accepted tools used in previous studies (3, 5). The final questionnaire was evaluated and approved by an independent evaluator.

The final questionnaire consisted of 12 questions, which concerned both patient follow-up and user feedback, Table 1. The questions about follow-up concerned the referrer’s opinion about whether the eventual outcome would be beneficial for the patient. The questions about feedback concerned the referrer’s opinion about whether the process was satisfactory (e.g., the way that the referral had been handled in the telemedicine network) and what the benefits were, for the patient and doctor.

**Table 1:** **Progress report questions**.

Question	Question type
(1) Was the case sent to an appropriate expert?	Feedback (S)
(2) Was the answer provided sufficiently quickly?	Feedback (S)
(3) Was the answer well adapted for your local environment?	Feedback (S)
(4) Were you able to follow the advice given?	Feedback (B_d_, B_p_)
(5) If NO, could you explain briefly why not	Feedback (B_d_, B_p_)
(6) Did you find the advice helpful?	Feedback (B_d_, B_p_)
(7) If YES, did it (tick any that apply)	
- Clarify your diagnosis?	Feedback (B_d_, B_p_)
- Assist with your management of the patient?	Feedback (B_d_, B_p_)
- Improve the patient’s symptoms?	Follow-up
- Improve function?	Follow-up
- Any other reason? Please specify	Follow-up/feedback
(8) Do you think the eventual outcome for the patient will be beneficial for the patient?	Follow-up
(9) Was there any educational benefit to you in the reply?	Feedback (B_d_)
(10) Was there any cost-saving as a result of this consultation? (tick any that apply)	Feedback (B)
- Saving for the patient/family?	Feedback (B_p_)
If YES, please explain briefly	Feedback (B_p_)
- Saving for the hospital/clinic?	Feedback (B_o_)
If YES, please explain briefly	Feedback (B_o_)
(11) Please add any other comments about this case specifically	Follow-up/feedback
(12) Please add any other comments about the service generally	Feedback (S)

*The questions concern follow-up (patient outcomes) or user feedback. User feedback encompasses satisfaction with the service (S) and benefit to the patient (B_p_), the doctor (B_d_), and the organization (B_o_)*.

### Automatic request for information

Modifications were made to the telemedicine system so that automatic requests for progress reports were sent to every referrer at a pre-determined interval after a new case had been submitted. The request allowed the referrer to log in to the server and then provided a link for the referrer to respond to the questionnaire.

### Demonstration of feasibility

To demonstrate the feasibility of the proposed approach, automatic requests for progress reports were issued in respect of cases submitted in the MSF telemedicine network for a 9-month period starting in October 2013. An email request was sent automatically by the telemedicine system to each referrer exactly 21 days after the initial submission of the case. When the referrer completed the progress report, an email notification was sent simultaneously to the expert(s) involved in the case and to the case-coordinators.

### Analysis of responses

Responses to the requests were analyzed approximately 4 weeks after the final request had been sent. The free-text comments were examined and, based on a content analysis, the main themes were extracted.

## Results

### Analysis of responses

During the pilot trial, 201 requests for progress reports were issued by the system and these elicited 41 responses from referrers (a response rate of 20%). The responses were largely positive. For example, excluding the Do not know and Missing responses, 95% of referrers stated that they found the advice helpful, 90% said that it clarified their diagnosis, and 94% said that it assisted with management of the patient. In addition, 95% said that the telemedicine response was of educational benefit to them. The responses are summarized in Table 2.

**Table 2:** **Summary of 41 responses**.

	Missing	Do not know	No	Perhaps	Yes	Yes (% of definite responses)	
(1) Was the case sent to an appropriate expert?		4			37	100	
(2) Was the answer provided sufficiently quickly?		6			35	100	
(3) Was the answer well adapted for your local environment?	1		8		32	80	
(4) Were you able to follow the advice given?			15		26	63	
(5) If NO, could you explain briefly why not							16 comments
(6) Did you find the advice helpful?		2	2		37	95	
(7) If YES, did it (tick any that apply)							
- Clarify your diagnosis?	12		3		26	90	
- Assist with your management of the patient?	9		2		30	94	
- Improve the patient’s symptoms?	15		16		10	38	
- Improve function?	15		16		10	38	
- Any other reason? Please specify							16 comments
(8) Do you think the eventual outcome for the patient will be beneficial for the patient?		8	3	14	16	48	
(9) Was there any educational benefit to you in the reply?	1		2		38	95	
(10) Was there any cost-saving as a result of this consultation? (tick any that apply)							
- Saving for the patient/family?	2	5	22		12	35	
If YES, please explain briefly							11 comments
- Saving for the hospital/clinic?	10	4	14		13	48	
If YES, please explain briefly							12 comments
(11) Please add any other comments about this case specifically							18 comments
(12) Please add any other comments about the service generally							17 comments

The qualitative analysis of the free comments confirmed this positive feedback from the responders, see Table 3. The expert advice was considered by the referrer as “clear, comprehensive, and useful,” helping both in the clinical management (diagnosis and management) and the information delivered to the patient and relatives. Referrers considered that the non-availability of an investigation or treatment that had been suggested was the main limitation in following the advice received. For this reason, some referrers emphasized the importance of making the expert aware of the constraints of the referral setting and the limited resources available.

**Table 3:** **Main themes in the free-text responses**.

Question	No. of answers	Type of comments	Main themes, with the number of recurrences in parentheses
Q5. If you could not follow the advice given, could you explain briefly why not	16	Main points	Investigation not available (5)
			Treatment unavailable (3)
			Inability to perform investigation (2)
			Disagreement on expert diagnosis (2)
			Discharged against medical advice (2)
			Cost not affordable by patient
			Patient lost to follow-up
			Advice not appropriate
			Not applicable
Q7e. Any other reason that you found the advice helpful	16	Main points	Diagnosis clarified or confirmed (2)
			Differential diagnosis discussed (2)
			Helpful discussion about diagnosis and management (2)
			Triggered decision to transfer patient to specialist (2)
			Confidence in experienced specialist
			Advice “clear, comprehensive”
			Useful information about disease (nature, management, complication signs) for patient and relatives
			Technical advice about how to take an X-ray
			Support in CT scan interpretation
		Other comments	Patient left against medical advice
			Difficulties in implementing treatment advised (e.g., chronic disease)
			Treatment still in progress: too early to assess
			Not applicable
Q10b. If there was a saving for the patient/family, please explain briefly	11	Main points	Avoid unnecessary referral to capital (4) because diagnosis given or chronicity of disease confirmed
			No further need for the patient to consult local specialists, saving both money and time (3)
			“Best diagnosis” obtained
			Clear information given to family and patient
			Avoid unnecessary harmful treatment or costly hospitalization
			Early referral suggested for congenital cardiac disease (preventing further complications)
			Specialized consultation not affordable by patient
Q10d. If there was a saving for the hospital/clinic, please explain briefly	12	Main points	Avoid unnecessary referral to specialist (3)
			No need to send investigation for interpretation (3)
			Avoid unnecessary and costly investigation
			Ambulatory management avoiding costly hospitalization
			Strengthened local staff decision to avoid costly referral
			Clear information helped management
			Not applicable (2)
Q11. Please add any other comments about this case specifically	18	About patient outcome	Patient lost to follow-up (making evaluation difficult), patient left, patient died
		About advice	“Very helpful” both for diagnosis and patient information, “excellent,” “very practical and realistic advice with our set up”
			Helpful for X-ray interpretation
			Useful guidance for specialized treatment
		About case	Critical cases with ICU transfer (2)
			Difficult case, but a feeling to have “offered everything we can”
			Difficult case, but a feeling that “comments improved both patient management and staff knowledge”
			Specialized surgical treatment performed
		To be improved	Problem of implementing expert advice in limited resource settings
			More detailed X-ray interpretation for educational purposes
			X-ray interpretation not appropriate
			Difficult to upload a large file to the server
			Expert to be better informed about limited resource settings to adapt better their advice
			Appropriateness and usefulness of expert advice improved after several emails (from Eurocentric – further investigations and management recommended – to field centered)
Q12. Please add any other comments about the service generally	17	Service	“Excellent” (3)
			“Very rapid and extremely useful to the field and consequently the patients”
			“Very useful – practical and informative”
			“Appreciated a lot” (2), “appreciated really”
			“Important with benefit for both client and medical personnel”
			“Effective” because quick answer
			“Very good quality and helpful”
			“Is the best”
			“Really quick with the best of ideas”
			“Good quality and very quick”
			“Advice adapted to MSF environment”
			“Good way of communication”
		Other comments	Using email instead of the telemedicine system has delayed the expert advice
			A delay in getting the answer reduces the benefit of expert advice
			Headquarters’ support is appreciated
			“Helpful to have opinions from different specialists on submission of one case”
			“It is great to be able to have expert advice in a very short time. It helps a lot to evaluate better and to make the right decisions for unknown diseases/symptoms. Great, great thanks”

*Note that one answer may include more than one theme*.

Satisfaction with the system was also very high and the words used by responders emphasized the efficiency of the system (“excellent, very good quality, quick, practical …”). In terms of benefit, avoiding unnecessary referral to a higher level of health care or avoiding further specialized consultation were mentioned as the main reasons for cost savings.

### Analysis of non-responses

During the pilot trial, questionnaires were completed for 41 cases. That is, no questionnaire was completed for the other 160 cases. These two groups of cases might have differed in some way, and any difference might be a reason why the referrers decided to respond or not to respond. Various characteristics of the two groups were, therefore, compared. The median age of the patients in Group 1 (those with responses) was 27.5 years, and the median age of the patients in Group 2 (those without responses) was 22.0 years. However, the difference was not significant (*P* = 0.13). There were no significant differences in the gender of the patients in the two groups, nor the type of queries required to answer them, nor the number of queries for each case, see Table 4.

**Table 4:** **Characteristics of the cases**.

	With reports (*n* = 41)	Without reports (*n* = 160)	*P*-value
Median age, years (IQR)	28 (9 −37)	22 (4 − 35)	*Z* = −1.5, *P* = 0.13
Number of patients
Young*	14 (35%)	70 (45%)	chi^2^ = 3.1, *P* = 0.21; *P*-value for trend = 0.12
Adult	23 (58%)	80 (52%)	
Older	3 (8%)	4 (3%)	
Gender	22 M, 19 F	77 M, 77 F	chi^2^ = 0.2, *P* = 0.68
Type of queries
Internal medicine	27 (34%)	89 (28%)	chi^2^ = 4.5, *P* = 0.34
Pediatrics	15 (18%)	96 (30%)	
Radiology	20 (25%)	71 (22%)	
Surgery	14 (18%)	45 (14%)	
Other	4 (5%)	19 (6%)	
No. of queries per case
1	20 (49%)	59 (37%)	chi^2^ = 7.1, *P* = 0.13; *P*-value for trend = 0.82
2	10 (24%)	64 (40%)	
3	5 (12%)	23 (14%)	
4	5 (12%)	7 (4%)	
≥5	1 (2%)	7 (4%)	

*Age groups defined as: young 0–17 years; adult >17–60 years; older >60 years.

### Responders and non-responders

Six referrers provided progress reports for every request they received, see Table 5. However, the majority provided either some reports, or none, see Table 6. There were no obvious differences between the three groups (responders to all, some, or none of the requests) in the characteristics available for comparison, see Table 7.

**Table 5:** **Referrers who provided progress reports for all requests**.

Referrer ID no.	Country	No. of progress reports provided	% Answered
1275	Chad	4	100
2444	Uganda	1	100
2491	Australia	1	100
2323	Germany	1	100
2475	Switzerland	1	100
368	Yemen	1	100
	*Total*	*9*	

*Note that some cases were submitted by headquarters staff on behalf of field doctors in low-income countries*.

**Table 6:** **Referrers who provided some or no progress reports**.

Referrer ID no.	Country	Unanswered requests		Answered requests	
		No. of requests	% Answered	No. of progress reports provided	% Answered
351	Cambodia	17	0	5	23
354	Kenya	16	0	3	16
180	South Sudan	12	0		
276	Tajikistan	8	0	2	20
356	Sudan	8	0		
254	South Sudan	8	0	1	11
211	Democratic Republic of the Congo (Kinshasa)	8	0	1	11
112	Uganda	7	0	1	13
298	France	6	0	1	14
1354	Myanmar, Burma	6	0		
2161	Central African Republic	5	0		
163	Ethiopia	5	0		
310	Democratic Republic of the Congo (Kinshasa)	5	0	4	44
2170	Democratic Republic of the Congo (Kinshasa)	5	0	1	17
1263	South Africa	4	0	1	20
1274	Chad	3	0	1	25
345	South Sudan	3	0		
2459	Democratic Republic of the Congo (Kinshasa)	3	0	1	25
315	Malawi	2	0	2	50
2478	Jordan	2	0		
75	Pakistan	2	0		
1279	Guinea	2	0	4	67
193	Papua New Guinea	2	0	1	33
2019	Syria, Syrian Arab Republic	2	0		
2480	South Sudan	2	0		
335	Sierra Leone	2	0	1	33
1356	Syria, Syrian Arab Republic	1	0		
2167	Democratic Republic of the Congo (Kinshasa)	1	0		
2163	Central African Republic	1	0		
1352	Swaziland	1	0		
2428	Spain	1	0		
2445	Afghanistan	1	0		
2476	Mozambique	1	0		
1222	Yemen	1	0		
2423	Central African Republic	1	0		
1258	Kyrgyzstan	1	0		
2455	Myanmar, Burma	1	0		
2301	France	1	0		
2468	Democratic Republic of the Congo (Kinshasa)	1	0	2	67
2498	Uzbekistan	1	0		
129	Bangladesh	1	0		
2442	Canada	1	0		
	*Total*	*161*		*32*	

*Note that some cases were submitted by headquarters staff on behalf of field doctors in low-income countries*.

**Table 7:** **Characteristics of those responding to all, some, or none of the requests**.

	All	Some	None	*P*-value
No. of referrers	6	17	25	
No. of referrals	15	388	415	One-way ANOVA *F* = 0.77, *P* = 0.47
Mean referrals per doctor	2.5	22.8	16.6	
Sex
Male	2	2	3	Male vs female: chi^2^ = 0.5, *P* = 0.77; *P*-value for trend = 0.73
Female	1	3	3	
Unknown	3	12	19	M/F vs unknown: chi^2^ = 1.6, *P* = 0.45; *P*-value for trend = 0.25
Country of referrers
Low-income countries	3	12	16	chi^2^ = 5.1, *P* = 0.08; *P*-value for trend = 0.17
Proxy countries	3	1	3	
Msf regions
OCA	2	7	9	chi^2^ = 7.5, *P* = 0.48
OCB	0	3	2	
OCBA	0	2	7	
OCG	1	1	3	
OCP	3	4	4	

## Discussion

The present work shows that both patient follow-up data and user feedback information can be obtained in a telemedicine network, via an automatic questionnaire. In a 9-month pilot trial, there was a response rate of 20%. How can we interpret this response rate? In physician surveys conducted in industrialized countries, a response rate of say 50–60% would be considered normal (6, 7). However, there is little published data about the response rate in online surveys of doctors in developing countries, and even less about the response rate in online surveys of doctors concerning the use of telemedicine in developing countries. A reasonable comparator is the study by Zolfo et al., of health-care workers using store-and-forward telemedicine in the management of difficult HIV/AIDS cases, which had a response rate of 19% (8).

The dangers of a low response rate are non-response bias (if the answers provided by respondents differ from the potential answers of those who do not answer), and response bias (if respondents tend to give answers that they believe that the questioner wants). Analysis of the characteristics of the referrers who did not respond, and the cases, did not suggest anything different about them in comparison with referrers who did respond. The comparison of referrers was, however, limited by the restricted information available about them. For reasons of information security, the telemedicine system stores little personal information about the users, and the accounts tend to be used by more than one person as staff are rotated through the field.

We were not able to identify obvious factors associated with a failure to respond. The response rate may, therefore, simply reflect the pressures of working in low-resource settings, and especially, the high turnover of field staff, which acts against the treating doctor being in post when a request for follow-up data is made some weeks later.

Measures to increase survey response rates are reasonably well understood, and include offering financial incentives, and following up online requests with copies of the survey sent out on paper. These are probably not appropriate in the present context. Nonetheless, it would seem prudent if this technique is to be adopted into routine service to try and increase the response rate. This raises a number of questions for future research:

1. when should follow-up data be requested? i.e., is 21 days the right time? Other work (2) suggests that a shorter interval, such as 1 week, would be appropriate, see Table 8
2. is there an optimum time interval for all patients, or does the optimum time vary, depending on the specialty being consulted?
3. what is the right number of questions? i.e., is 12 questions too many? Reducing the survey to 2–3 questions might make a response more likely.
4. is it appropriate to ask for user feedback each time that a follow-up report is requested? Should requests for user feedback be made separately from requests for follow-up data (and less frequently)?
5. is a single follow-up report sufficient, or should there be say a short-term and a longer term report?

**Table 8:** Data from a previous survey,* (A) responses from referrers; (B) responses from specialists.

	Yes/multiple choice	No	Unknown	Total answered	Skipped	Majority response
**(A)**						
**Question to referrer**
Q37: Did you give the specialist any feedback about the patient?	41%	59%	–	34	31	No 59%
Q38: If no, was it because …		NA	NA	37	43	Lack of time 30%
-Patient lost to follow-up	14	
-Lack of time	30	
-Forgotten to update	24	
-Feeling it was not necessary	16	
-Worse outcome or patient died	3	
-Difficulties with Internet access	14	
Q39: Do you think that feedback about the patient is.		NA	NA		27	Desirable 43%
-Optional	14	
-Desirable	43	
-Necessary	30	
-Mandatory	14	
Q40: In your opinion, is the patient likely to be available for follow-up in 2–4 months?	22%	46%	32%	37	27	No 46%
Q41: In your opinion, when would it be relevant to give follow-up information? (i.e., completing a progress report)		NA	NA	38	28	After 1 week 53%
-After 1 week	53	
-After 2 weeks	24	
-After 1 month	18	
-After 3 months	5	
-After 6 months	0	
**(B)**						
**Question to specialist**
Q37: Did you receive any follow-up information about this patient?	8%	92%	–	63	36	No 92%
Q38: Do you think that feedback about the patient is.		NA	NA	67	32	Desirable 52%
-Optional	1	
-Desirable	52	
-Necessary	29	
-Mandatory	18	

*Data from the MSF survey (50 questions) sent to 294 referrers and 254 specialists (in French and English) in December 2013 (2).

As mentioned in the Introduction, it is highly desirable to obtain follow-up data for each case. Even though there are other ways to obtain follow-up information, e.g., from the regular dialog between expert and referrer, the benefit of using an automatic request is that a standardized report is obtained for each case. Thus, the main problem in practice is the low response rate, and how best to encourage the referrer to complete the questionnaire. One potential way to increase the response rate would be to reduce the number of questions, in order to allow the referrer to answer within 1–2 min. As shown in a previous survey (2), the main reasons given for not answering were a lack of time > forgotten to update > patient lost to follow-up > difficulties with Internet access (Table 8). This is why we propose to separate the reporting of follow-up data from obtaining user feedback.

If user feedback is solicited separately from the follow-up data, then a natural time to request it would be when the monthly quality assurance (QA) review is conducted (9). This activity involves an expert panel making an assessment of a recent case that has been selected at random. If user feedback is requested from the referrer for the same case, then both the panel’s and the referrer’s views on the quality of the teleconsultation can be compared.

Finally, it is worth noting that specialists tend to underestimate the value of their responses. In a recent survey (3), Patterson examined the perceived value of telemedicine advice. There were 62 cases where it was possible to match up the opinions of the referrer and the consultants about the value of a specific teleconsultation. In 34 cases (55%), the referrers and specialists agreed about the value. However, in 28 cases (45%), they did not; specialists markedly underestimated the value of a consultation compared to referrers. A survey of MSF telemedicine users found a similar phenomenon (2). This reinforces the importance of obtaining user feedback from the referrers, who are best placed to evaluate the benefits to the patient.

## Conclusion

Obtaining data from referrers by automatic request is feasible. The technique provides useful information for specialists and for those running the network. The modest response rate could be improved. Since obtaining follow-up information on each case is essential to best practice, a proposal to improve the response rate is to re-design the follow-up questionnaire to be as simple as possible, and to obtain user feedback separately, by sending a more detailed questionnaire in parallel with the randomly selected cases reviewed each month by the QA expert panel.

## Conflict of Interest Statement

The authors declare that the research was conducted in the absence of any commercial or financial relationships that could be construed as a potential conflict of interest.
